# Supplementary material for: A Mathematical Model of the Phosphoinositide Pathway
Source: Sci Rep. 2018 Mar 2;8:3904. doi: 10.1038/s41598-018-22226-8 (PMC5834545; doi:10.1038/s41598-018-22226-8)
Supplement: Supplementary file 1 — Supplementary Information [file 41598_2018_22226_MOESM1_ESM.pdf]

Supplements:

A Mathematical Model of the Phosphoinositide Pathway

Daniel V. Olivença, Inna Uliyakina, Luis L. Fonseca, Margarida D. Amaral, Eberhard O. Voit, Francisco R. Pinto

1. SUPPLEMENTARY METHODS AND RESULTS

1.1. Background

We assume that phospholipids may move freely across the cell membrane and that phosphoinositides are in the inner leaflet of the plasma membrane, as reported by van Meer [1] and Fadeel [2]. Model reactions take place at the inner leaflet of a 1 μm^2 patch of plasma membrane and in an adjacent region of the cytoplasm, with a height of 0.01 µm. We assume that influxes and effluxes of lipids by diffusion from or to adjacent patches are balanced. Within this 3D environment, we suppose that the enzyme kinetics in the model follow generalized mass action processes. We do not account for the binding of cytoplasmic enzymes to the membrane prior the initialization of their catalytic activity.

1.2. Model Design

1.2.1. Transport fluxes

Transport fluxes reflect vesicle and non-vesicle-mediated transport [3–5]. It is unclear what percentage of the total transport is vesicle-independent. The transport can be accomplished by specialized proteins, called lipid transport proteins (LTPs), or can happen spontaneously at membrane contact sites (MCSs). We assume that these effluxes have first-order kinetics ($f_{i\rightarrow}$ = 1) and have one common rate constant. This last assumption is not trivial. For example, some species of phosphoinositides are necessary for the initiation of vesicle formation so they might have a slightly higher exit rate. At the same time, non-vesicle mediated transport is facilitated by LTPs, and LTPs with varying affinities for different phosphoinositides are presently not known. Overall, we do not possess enough information to include different efflux rate constants for each model variable.

Alberts *et al.* [6] reported that a macrophage ingests 3% of its plasma membrane per minute, while a fibroblast only ingests 1%. As a compromise, we set the amount of internalized plasma membrane due to endocytosis as 2% per minute. We added 2.5% for non-vesicle transport, resulting in a total of 4.5% per minute. The input flux values were restricted in order to allow 4.5% of the membrane phosphoinositides to recycle per minute. This setting made the levels of PI robust to alterations in other phosphoinositide pools. We also tested a level of 1% per minute. The model exhibited a similar behaviour, but the PI pool fluctuated considerably in response to alterations in other phosphoinositide pools. For example, manipulations in the PI(4)P and PI(4,5)P2 levels caused an undue increase of more than 15% in the PI pool. Of course, it is imaginable that such fluctuations could exist and have a physiological meaning, for instance, to

signal to the kinases responsible for PI(4)P and PI(4,5)P2 production that these lipids are being depleted. However, we found no reports in the literature of this phenomenon, and therefore chose the model setting corresponding to a more stable PI pool.

1.3. Parameter Estimation

The phosphoinositide literature is vast. However, many experimental results are presented in ways that are difficult, if not impossible, to translate into numerical values of model variables or relative changes. Other publications present results that involve manipulations or perturbations of genes or proteins that are not included in our model. In the end, the experimental data used in the following to parameterize the model consist of all data from the literature that permitted reliable translation into model variables or changes in model parameters.

Each flux $v_{i\rightarrow j}$ contains three parameters, namely a rate constant $\gamma_{i\rightarrow j}$, one or more kinetic orders $f_{i\rightarrow j}$, and the available quantity of enzyme that catalyses the reaction in this flux ($E_{i\rightarrow j}$). These three types of parameters need to be estimated to populate the model equations.

If parameter values are found in the literature, they are typically given as K_m and specific activity values. Many of such values have been collected in the database BRENDA [7]. While BST does not use these parameters, they are easily converted into values for rate constants and kinetic orders of GMA model [8]. Enzyme kinetic parameters retrieved from literature and BRENDA are shown in Table ST4.

K_m and specific activity values collected in BRENDA are in mM and µmol/min/mg respectively. Before using these parameters in the estimation of rate constants and kinetic orders, K_m's were converted to molecules/μm^2 and the specific activities to molecules/min/mg. While converting volume units to area units, we considered that the number of molecules present in a 1 μm^2 membrane patch is the same number present in a 1 μm^3 volume including the membrane patch and a thin layer of surrounding cytosol.

Table ST4 also presents the known cellular localization of each enzyme as retrieved from UNIPROT annotations and described by Sasaki *et al.* [9]. Since the model aims to simulate a patch of the plasma membrane, enzymes should be considered if they are present in the cytosol or in the plasma membrane. This is true for all enzymes except the three SAC phosphatases. However, there is evidence that these phosphatases can control directly the levels of PI4P at the plasma membrane by acting at membrane contact sites between the ER and the plasma membrane [10].

For enzymes that are present in multiple cellular compartments it is difficult to assess their relative contributions to the catalysis of a given reaction in each of the compartments. This lack of information is not critical in our approach since the parameters defining the amount of enzyme catalysing each flux in the plasma membrane patch ($E_{i\rightarrow j}$) are numerically adjusted to optimize the model fit to the experimental data.

The parameters in Table ST4 could not directly be applied to the model since there is no one-to-one relationship between each enzyme and each flux. Some enzymes catalysed more than one flux and many fluxes may be catalysed by more than one enzyme. Because quantitative details

regarding these multiple processes are lacking, enzymes catalysing the same reaction were grouped using information in Balla [11] and Sasaki *et al.* [9].

For each group, one enzyme was chosen to define the flux parameters. The chosen enzyme was normally the one identified in the literature as the main catalyst of the respective flux. The enzymes and associated fluxes are presented in in Table ST5 for kinases and in Table ST6 for phosphatases.

Generally, more information is available for kinases than for phosphatases. Moreover, there is a difference concerning the assignment of kinases and phosphatases to different fluxes. As Delage *et al.* [12] observed, kinases are often specific while phosphatases are polyvalent. Of the ten fluxes catalysed by phosphatases, seven can be catalysed by synaptojanins (SYNJ) and five by suppressor of actin (SAC) [9]. This redundancy is in stark contrast with the kinases that catalyze one or two reactions at most. An exception is phosphoinositide 3-kinase (PI3K), which is polyvalent if one allows for primary and secondary reactions. It is also noteworthy that PTEN, probably the most studied phosphatase as a consequence of its role as tumour suppressor, is specific for PI(3,4,5)P3 and PI(3,4)P2 substrates [13].

Fine-tuning of the model was accomplished by adjusting the levels of enzymes ($E_{i\rightarrow j}$) and fluxes entering and exiting the system ($\gamma_{\rightarrow 0}$, $\gamma_{\rightarrow 3}$, $\gamma_{\rightarrow 4}$, $\gamma_{i\rightarrow}$). These parameters were manually tuned until the model reached a steady state where phosphoinositide levels agreed with values reported for the cell membrane (Table ST1) and model simulations replicated observed phenomena (Table ST2 and Fig. 3). Most of the remaining parameters, that is, rate constants and kinetic orders derived from the literature were not altered. PIP5KII parameters had to be adjusted to allow a steady-state composition compatible with literature values. This enzyme catalyses the flux $v_{5\rightarrow 45}$ [11] [9]. Using the original parameters for this enzyme would make $v_{5\rightarrow 45}$ rather slow, yielding an accumulation of PI(5)P at around 700 molecules per μm^2, which is outside the intervals reported in the literature. One could slow down the sources of PI(5)P in order to obtain a smaller pool, but that would reduce the turnover of PI(5)P which is supposed to be high [11], and the model would no longer replicate the observations regarding PI(5)P, PI(3)P and PI(3,5)P_2 documented in the literature. Further, using the reported PIP5KII parameterization found in the literature [14], PI(5)P and $v_{5\rightarrow 45}$ would not sustain the PI(4,5)P_2 pool. We also had to re-estimate two rate constants, $\gamma_{3\rightarrow 35}$ and $\gamma_{35\rightarrow 5}$. Values retrieved from BRENDA were too small to replicate observed phenomena. For example, Bulley *et al.* [15] stated that the majority of the PI(5)P pool is formed from PI(3,5)P2. With the values retrieved from the literature [16] $v_{0\rightarrow 5}$ would have a greater contribution to the PI(5)P pool than $v_{35\rightarrow 5}$. Increasing $\gamma_{35\rightarrow 5}$ forced us to increase $\gamma_{3\rightarrow 35}$, which subsequently increased the PI_Kfyve activity when PI(3)P was used as substrate rather than PI. The increase of $\gamma_{35\rightarrow 5}$ could be explained by a regulation that is not implemented in the model: the activation of MTMRs by PI(5)P [17]. The amount of PIP5KII in the model is the highest as kinases are concerned. There is evidence in the literature of higher abundance of PIP5KII than PIP5KI [18].

After the manual adjustments, as described above, a genetic algorithm was used to search for a parameter set that produced the closest fit to a set of observed phenomena.

The implemented genetic algorithm has two mechanisms to create diversity: mutation and recombination. The mutated parameters are obtained multiplying the initial value by a random value from a normal distribution of mean 1 and standard deviation of 0.3 or 0.5. Each new parameter set is only accepted if a set of validity conditions is met (Table ST7). Higher standard deviation values would make the algorithm run very slowly due to a high number of invalid sets. Recombination combines two parameter sets and thereby generates a new set where each parameter is the average of the corresponding parameters in the two parent sets.

Each generation starts with 10 distinct parameters sets. In the first generation, the initial set consists of the manual solution and 9 viable mutants are obtained from this parent set. All pairs of these 10 sets are combined producing 45 recombined sets. Additional 10 parameter sets are created by mutating all enzyme levels, inputs and outputs parameters from 10 of the existing 55 sets picked randomly. Next, 35 new sets are created recombining a random mutant with one of the previous 55 sets. The resulting 100 sets are the source for further 100 minor mutant sets that alter between 1 and 5 parameters of the 100 existing sets. The final 200 parameter sets are then scored and the 10 sets with the best score are selected to start the next generation. The criteria for scoring the sets are presented in Table ST7.

Two versions of the genetic algorithm were employed: one where the 10 initial progenitor sets of each generation could be part of the next generation and one where they were excluded. The former algorithm converged rapidly but the latter found best scores more quickly. In the long run, both algorithms delivered sets with similar scores that were 50% better than the manually determined set. Final parameters for the fluxes and protein amounts per μm^2 are shown in Table ST3.

1.4. Sensitivity Analysis

Local sensitivity analysis was implemented as described in Chen *et al.* [8] and combined sensitivities, also known as global sensitivities, were computed as detailed in Kent *et al.* [19].

Parameter sensitivities were assessed numerically by increasing each parameter, one at a time, by 1% and computing the new steady state of the system. When the relative change in the steady-state value of a dependent variable is higher than 1% (or lower than -1%) the sensitivity indicates that a change in the parameter value is amplified in the steady-state of the dependent variable. Smaller sensitivities indicate attenuation of a perturbation. Although there are exceptions, most biological systems are expected to show sensitivity values between about -3 and +3. However, in signalling systems, the sensitivities may be much higher.

The model contains 64 parameters but the sensitivity analysis can be reduced to 46 parameters, because there are 21 pairs of rate constants ($\gamma_{i \rightarrow j}$) and a corresponding enzyme quantity ($E_{i \rightarrow j}$) that always appear as a product in a flux equation. Multiplying a constant to $E_{i \rightarrow j}$ or to the corresponding $\gamma_{i \rightarrow j}$ will give the same relative change in flux and, thus, in steady state. Consequently, the sensitivities for the rate constants are identical to the corresponding enzyme quantity.

The results of the sensitivity analysis are conditional on the parameter set that provides realistic steady-state values and replicate the observed phenomena. Furthermore, they reflect responses to changes in individual parameters. However, multiple small or intermediate errors in parameter values could collectively have a larger impact on the performance of the system. To address this question, combined sensitivities were studied using a Monte-Carlo approach. Specifically, we assigned a random uncertainty of ±5%, ±10%, ±20%, ±50% or ±100% for every parameter. With every combination, we created a new parameter set and recorded those combinations that led to a steady state in up to a simulation time of 1000 minutes. With this method, 5,000 new parameter sets were retrieved for each level of uncertainty. For each parameter set sampled we calculated the local sensitivities.

1.4.1. Sensitivity Analysis Results

The complete list of local sensitivities is presented in Table ST8. Only 20 out of 368 (~5%) computed sensitivities have an absolute value greater than 1. The highest sensitivity value indicates an increase of 5.7% in PI(3,4)P2 when $f_{345\rightarrow34}$ is increased by 1%. The parameter $f_{0\rightarrow3}$ has the highest number of high sensitivities, and these are related to PI(3)P, PI(5)P and PI(3,5)P_2.

The high proportion of low sensitivities shows that the model system is very robust to environmental and mutational challenges that perturb a parameter value. It also implies that small errors in model parameterization will not affect model behavior in a significant manner.

We intended to investigate whether the distributions of sensitivities might suggest a different behavior associated with a parameter change than the one suggested by the local sensitivities. Generally, if the sensitivity of a parameter exhibits large variability, it might suggest a lack of system robustness [19].

The combined sensitivity results suggest that most parameter sensitivities are concentrated closely around the local sensitivity value. 112 out of 368 sensitivities (30.4%) led to a low overall sensitivity, even if the parameters were allowed to vary up to ±100%. Of the 256 that presented higher combined sensitivities, only 42 exhibited this behaviour with uncertainties lower or equal than 50%.

Considering these results, we can conclude that small changes in parameter values do not alter the sensitivity profile of the dependent variables. These observations suggest that the model is robust with respect to uncertainties in parameters.

Studying the sets with 100% uncertainty we found that $\gamma_{i\rightarrow}$ (the rate constant of the effluxes of all pools) presented high sensitivities in all dependent variables. $f_{0\rightarrow4}$, $f_{45\rightarrow4}$, $f_{45\rightarrow345}$ and $f_{0\rightarrow45}$ presented high sensitivities in 7 dependent variables, $f_{45\rightarrow0}$ in six and $f_{4\rightarrow0}$, $f_{45\rightarrow5}$, $f_{345\rightarrow45}$, $f_{345\rightarrow34}$ and $f_{4\rightarrow34}$ in five. This result highlights the importance of the linear pathway of PI, PI(4)P, PI(4,5)P_2 and PI(3,4,5)P_3 in the model.

The result is consistent with the earlier traditional sensitivity analysis. All parameters that in the combined sensitivity study cause high sensitivities in five or more dependent variables are also

presented in the individual sensitivity analysis, except for $f_{45\rightarrow 0}$, $f_{0\rightarrow 45}$ and $f_{45\rightarrow 5}$. This suggests that the model was parameterized so that $v_{0\rightarrow 45}$ and $v_{45\rightarrow 0}$ fluxes have a small influence, although their potential for high sensitivities is considerable.

1.5. Identifiability Analysis

If a model is only slightly perturbed even in response to large changes in a parameter value, does it mean that the parameter value is correct? This question becomes complicated if the sensitivities of two or more parameters are correlated and the increase in one can be compensated with an alteration in one or more others. The issue is related to the existence of infinite parameter combinations that produce essentially the same model output. This redundancy is especially important if we want to suggest an experimental design to populate the model with parameters values. These issues are related with the problem of parameter identifiability, which is defined as the ability to identify the true value of a model parameter [20].

To find the best identifiable parameters we implemented the method described by Srinath and Gunawan [20] and Yao *et al.* [21], which is based on the local sensitivity matrix. For each column of the matrix, the Euclidian norm is calculated and the column with the highest magnitude is selected. If the magnitude exceeds a certain threshold, the parameter corresponding to this column is identifiable. This column is removed from the local sensitivity matrix. The projection of the removed column on the remaining columns is computed and subtracted from them. This procedure creates a new local sensitivity matrix. The process is repeated until the highest magnitude is below the threshold. All remaining parameters are considered non-identifiable.

1.5.1 Results of the Identifiability Analysis

In the phosphoinositide pathway model, the best-identifiable parameters are $f_{4\rightarrow 0}$, $f_{3\rightarrow 35}$, $f_{5\rightarrow 45}$, $f_{45\rightarrow 345}$, $f_{35\rightarrow 5}$, $f_{4\rightarrow 34}$, $f_{45\rightarrow 4}$ and finally $\gamma_{i\rightarrow}$ (Fig. SF1d). These parameters constitute a subset of those parameters that exhibited high local sensitivities. The parameters that were considered non-identifiable either have very small sensitivities, or their effect on the dependent variables may be replicated by a linear combination of perturbations in identifiable parameters.

1.6. Monte-Carlo Exploration of the Parameter Space

The identifiability analysis suggests the existence of alternative parameter sets that replicate both, the reported steady-state levels of phosphoinositides and the observed relationships between steady-state levels. To identify possible alternative parameter sets satisfying these conditions, we explored 79,993 random parameter sets using a Monte Carlo approach. Only the most identifiable parameters (discussed in the previous section) plus the input and output fluxes were allowed to vary between 50 and 150 per cent from their reference value. These parameter sets were only accepted if they reached a steady state in up to 5000 minutes of simulation time.

Non-identifiable parameters were not varied in order to optimize the parameter space exploration, because varying non-identifiable parameters would yield small changes in system behavior. Additionally, linear combinations of perturbations in identifiable parameters can mimic most of the effects of perturbations in non-identifiable perturbations. Input fluxes were also

varied although they were not identifiable. This variation was necessary to accommodate changes in effluxes ($\gamma_{i\rightarrow}$) and still allow for the system to achieve a steady state.

1.6. Results of the Monte-Carlo Exploration of the Parameter Space

Among the initial 79,993 combinations of parameter values, 79,985 reach a steady-state within 5000 minutes and 1452 have phosphoinositide levels within the intervals retrieved from the literature. Among these, 231 replicate the relative amounts between the phosphoinositide pools. Intriguingly, only 117 (the set used to parameterize the model and 116 alternatives), less than 0.15% of all surveyed sets, satisfy the previous conditions plus the fact that effluxes are less in magnitude than 7% and the influxes are less than 25% of the respective phosphoinositide pools. Indeed, the 116 alternative sets of parameters have outputs that are similar to the ones produced by the optimized parameter set but are less concordant with of the phenomena retrieved in the literature. For example, as can be seen in Fig. 5, taking the score of the manually found parameter set as a base score of 20, all of the 116 admissible alternative parameter sets have a higher score, i.e., present less concordance with the conditions of the scoring function.

The results of this analysis suggest that the information available is sufficient to restrict the parameter space to a region that is compatible with all experimental observations. The alternative parameter sets are characterized by alterations that cancel each other in the system's input of PI, PI(3)P and PI(4)P and output fluxes.

The conditions imposed to find alternative parameter sets are not very restrictive. For example, we accepted data sets with the levels of PI between 200,000 and 400,000 and for PI(4)P and PI(4,5)P_2 between 5,000 and 20,000. As such, the small number of parameter sets that satisfy all the tests was not due to narrow test conditions but a genuine scarcity of combinations of parameters that satisfy all pertinent phenomena reported in the literature.

It is impossible to discern which set is the best representation of reality, not only because there are gaps in information, like missing measurements of the input fluxes, but also because these parameter sets can correspond to different cell types, states or membrane configurations. However, the initial parameter set used in the model has kinetic parameters in accordance with the literature and steady-state values that are closest to the center of the reported intervals for phosphoinositide levels.

1.6. Validity Test Using an siRNA Knockdown

1.6.1. Preparation of siRNA coated multi-well plates

Multi-well plates (384-well plates) (BD Falcon #353962) were coated with customized siRNAs (Silencer® Select, Ambion) for solid-phase reverse transfection adapted from a previously reported protocol (Erfle H et al., 2007). An aqueous 0.2% (w/v) gelatine solution was prepared and filtered with 0.45µM pore size filter and a 0.4M glucose solution was prepared in Opti-MEM (Gibco #51985). Then, a transfection mix was prepared by mixing 1.662mL of the sucrose/Opti-

MEM solution, 969µL of Lipofectamine® 2000 (Gibco #12566014) and 969µL doubly distilled water. This transfection mix was distributed into a 96-conic well plate (35µL/well, “Plate A”). In parallel, fibronectin was diluted in the 0.2% gelatine solution to a concentration of 1%. This solution was distributed into another 96-conic well plate (96µL/well, “Plate B”). Then, 5µL of a 3µM siRNA solution and 7µL of the transfection mix (“Plate A”) were incubated in each well of a low volume 384 well plate (“Plate C”). After 20-min incubation, 7µL of the fibronectin solution (“Plate B”) were added. 3µL of the contents of each well in “Plate C” were diluted fifty fold in a 384 deep well plate using doubly distilled water. Finally, 15µL of each well were transferred to a 384-well imaging plate, lyophilized and stored in an anhydrous atmosphere before cell seeding.

1.6.2. ENaC microscopy-based live-cell functional assay

1.6.2.1. Preparation of the functional assay

The live-cell assay used to identify the novel regulators of ENaC activity constricted on transfecting A549 cells with different siRNAs by solid-phase reverse transfection (previously described in the section *Preparation of siRNA coated multi-well plates*). Cells were plated in 384-well plates containing different human siRNAs (2500 cells per well) and incubated for 48h or 72h at 37ºC with 5% CO_2. Afterwards, cell nuclei were stained for 1h with Hoechst-33342 dye (Sigma-Aldrich #B2261) (1/10000) diluted in Ringer solution (145mmol/L NaCl, 0.4mmol/L KH_2PO_4, 1.6mmol/L K_2HPO_4, 5mmol/L D-glucose, 1mmol/L $MgCl_2$ and 1.3mmol/L Ca-gluconate) (30µL/well) at 37ºC. After washing with Ringer solution, cells were incubated for another 10min with diluted voltage-sensitive FLIPR® Membrane Potential Assay (FMP) (Molecular Devices, #R8042) staining solution (20µL/well) at 37ºC in the pre-warmed and humidity saturated (50-70% of humidity) microscope chamber of an automated epifluorescence Scan^R screening microscope (Olympus Biosystems), comprising a cooled 12 bit 1344x1024 pixel resolution C8484 CCD camera (Hamamatsu), Marzhauser SCAN IM IX2 scanning stage, metal halide light source (MT20), standard filter sets and an automated liquid dispenser.

1.6.2.2. Image Acquisition

The first row of the cells in the 384-well plate was imaged in the wide-field Olympus Scan^R microscope with a 10x objective (Olympus, UPSAPO) in the Cy3 channel and DAPI channel with an exposure time of 5ms and coarse auto-focus (two images per well, 5min/row). Then FMP containing 30µM amiloride hydrochloride (Sigma #A7410) was added by an automatic liquid dispenser adapted to the microscope stage. The image acquisition was initiated after 5min of incubation with amiloride.

1.6.2.3. Image Analysis

Overall transfection efficiency was assessed by observing if cells transfected with siRNAs compromising chromosome segregation exhibited mitotic phenotypes (Simpson et al., 2012). Failure to observe these phenotypes in more than 75% of images implied the rejection of the corresponding plate from analysis.

The intensity of FMP fluorescence (IF) before and after adding amiloride was quantified using the open source cell image analysis software CellProfiler. Nuclei were identified as primary

objects and FMP fluorescence measured in Cy3 channel as secondary object. The amiloride-sensitive fluorescence ratio for each cell was calculated applying the following formula:

$$Ratio = \frac{IF_{\text{before amil}} - IF_{\text{after amil}}}{IF_{\text{before amil}}} \times 100$$

Each image was corrected for the background and several quality controls were applied: cells with too high or too low intensity; abnormal area shape, eccentricity; low number of cells per well; cells out-of-focus, localized near the edges or that changed their position in the well were not quantified. The amiloride-sensitive ratios were further analysed in R in order to automatically identify the potential "hits", which are the ones whose ratio deviate more than two standard deviations from the ratio of the negative control, as calculated from the following formula:

$$Deviation = \frac{\overline{Ratio_{\text{siRNA}}} - \overline{Ratio_{\text{Negative control}}}}{2 \times SDM_{\text{Negative control}}}$$

Where SDM is a standard deviation of the mean and “scrambled” siRNA was used as a negative control.

We considered as significant Amil-sensitive ENaC functional effects, those whose magnitudes were larger than twice the negative control´s SDM. Therefore, we defined ENaC function enhancers as those conditions having a Deviation Score above +1 and ENaC function inhibitors as those having a Deviation Score below -1. Additionally, Student´s t-test was performed to quantify statistical significance versus the corresponding negative control.

2. SUPPLEMENTARY FIGURES

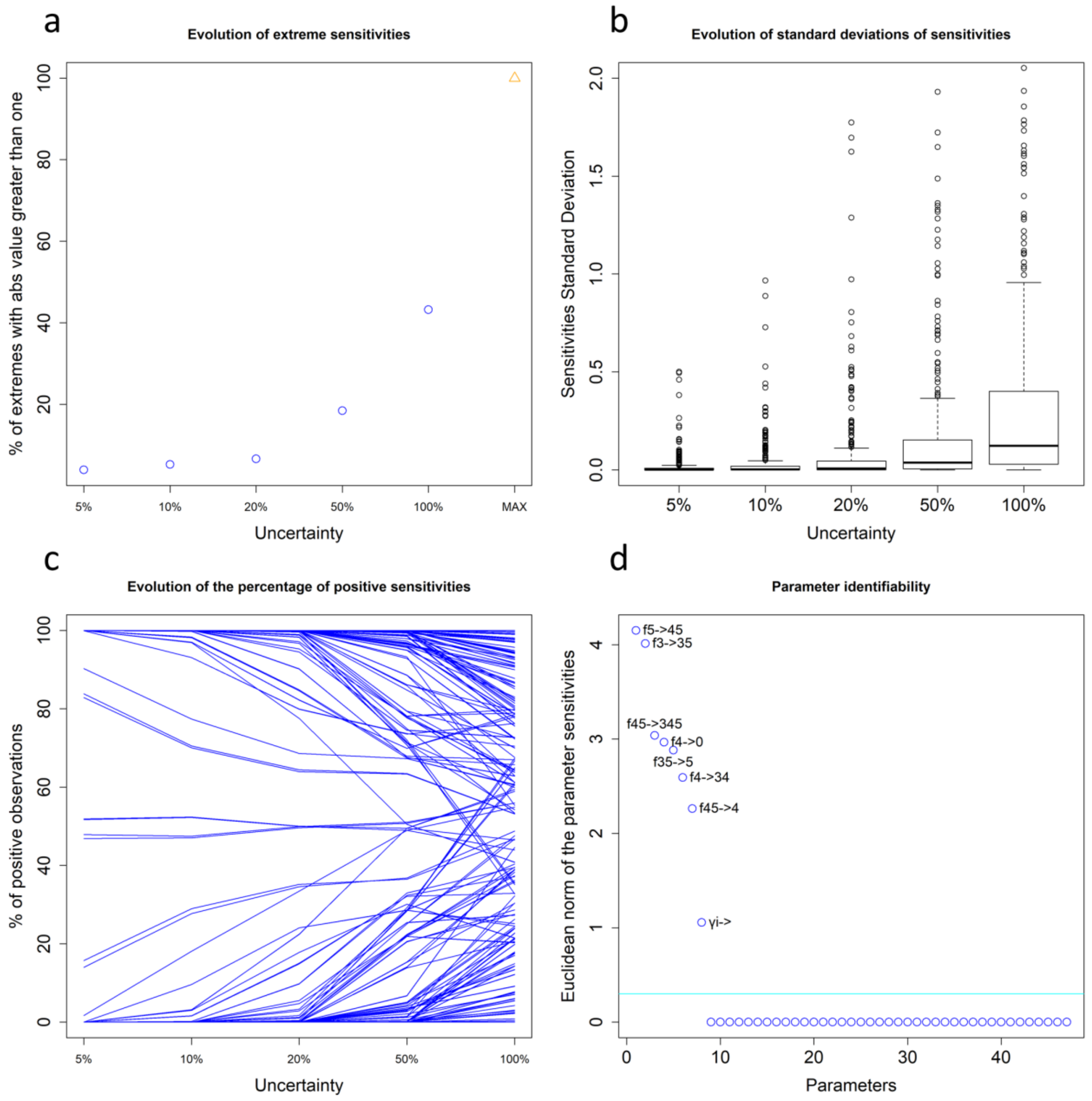

Supplementary Figure 1 (SF1) – Evolution of sensitivities due to an increasing level of uncertainty and parameter identifiability. a) Evolution of the number of extreme sensitivities with absolute values greater than 1 as a function of the level of uncertainty in parameter values. MAX corresponds to the total number of extremes in the model (736 extremes, which is twice the number of parameters). A noticeable increase begins around the 50% uncertainty level. b) Evolution of the standard deviations of sensitivities due to increasing uncertainty. The boxplot corresponding to 100% uncertainty does not show all outliers, the highest of which is 48.09. This finding suggests that the variance in sensitivity is moderate at least up to 50% uncertainty. c) Evolution of the percentage of positive sensitivities according to parameter value uncertainty.

Each line corresponds to one pair of a parameter and a dependent variable. For low levels of uncertainty, the majority of the observed sensitivities are almost all positive or all negative, which means that the sign of the sensitivities are constant for small perturbations. The reason for this consistency is that the background parameter sets lead to models with similar behaviour. The result also suggests that the model behaviour is consistent even under moderate uncertainty and only starts to vary for 50% or more uncertainty in parameter values. d) Plot showing the best-identifiable parameters. $\gamma_{i\rightarrow}$ represents for the rate constant for all exit fluxes. Graphs created in R[22].

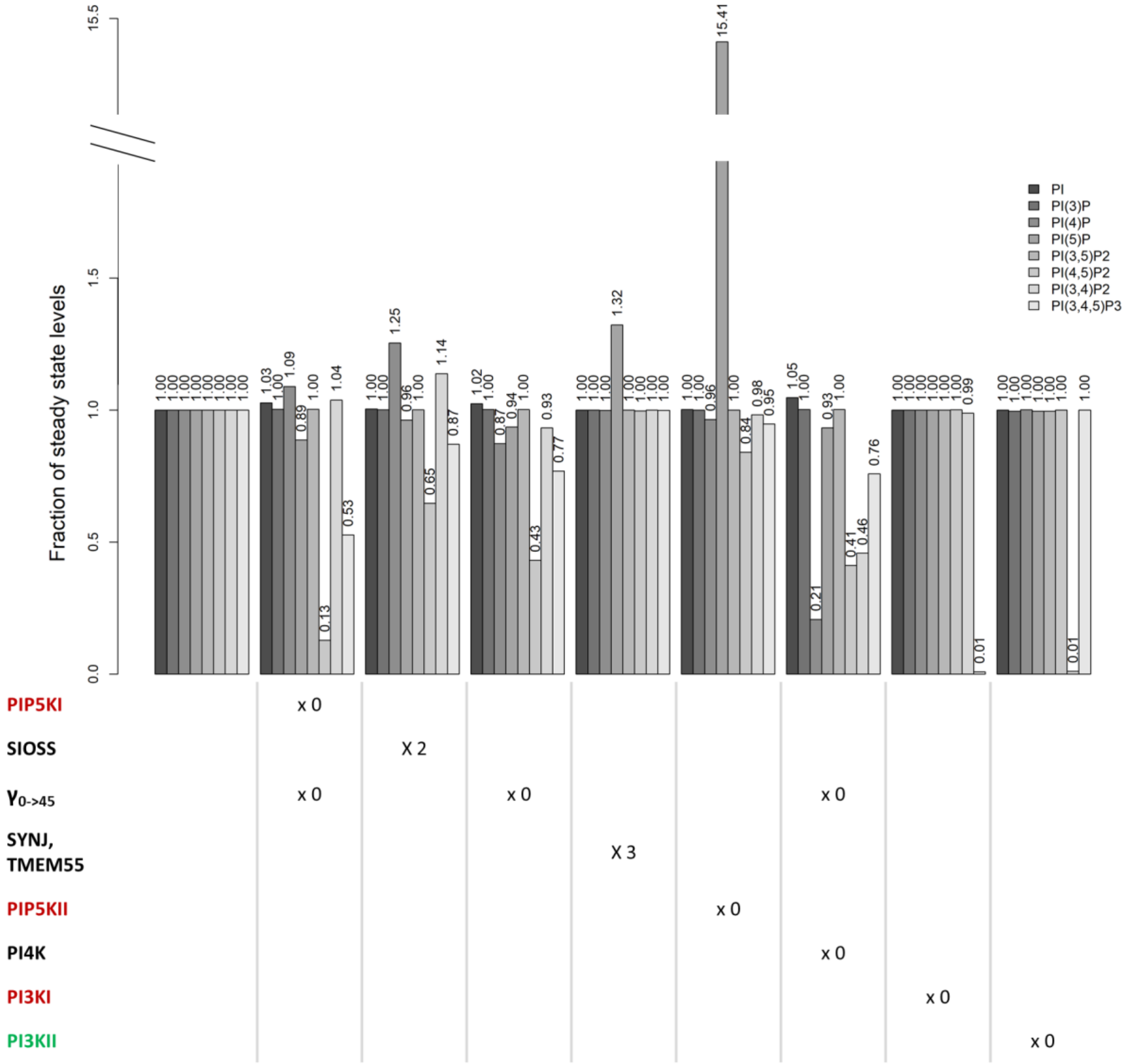

Supplementary Figure 2 (SF2) - Alterations in all pools of the model as a consequence of perturbations in enzyme activities. Changes in enzyme activity simulate the effect of an siRNA screen performed by Almaça and colleges[23]. Graph was created in R[22] and x axis labels added in PowerPoint.

3. SUPPLEMENTARY TABLES

Supplementary Table 1 (ST1): Levels of phosphatidylinositol and phosphoinositides in the membranes of mammalian cells and corresponding steady-state values in the model.

Molecule	(mol %) of total cellular inositol lipids	Molecules / μm²	Source	Model values at steady state
PI	90%	200000 – 400000	[11] [1] [24]	304372.1
	80%	40000 - 160000	[25]	
PI(3)P	0.36%	800 – 6000	[11]	100.0
	0.05% - 1.5%	25 – 3000	[25]	
	0.2%	600	[26]	
	0.175%	525	[27]	
	0.3%	100	[13]	
PI(4)P	2,24%	5000 – 20000	[11] [28] [24] [25]	10040.1
	6%	18000	[26]	
	3%	9000	[27]	
PI(5)P	Very small	Very small	[11]	100.5
	0.5% - 2% of PI(4)P	75 – 300	[15]	
	4%	1200	[26]	
	.3%	900	[27]	
	0.1% – 0.5%	50 – 1000	[25]	
PI(4,5)P2	2,24%	5000 – 20000	[11] [28] [24] [25]	10001.0
	3%	9000	[26]	
	5%	15000	[27]	
PI(3,5)P2	7 to 50 fold less than PI(5)P	1 – 57	[29]	20.2
	0.1% - 1%	50 – 2000	[25]	
	0.055%	165	[26]	
	0.04%	120	[27]	
PI(3,4)P2	Very small	Very small	[11]	20.6
	0.1% - 1%	50 - 2000	[25]	
PI(3,4,5)P3	0.036%	80 – 1000	[11] [28]	639.5
	0.1% - 1%	50 - 2000	[25]	
Total	100%	220000 – 450000	[11]	325294.0
		50000 - 200000	[25]	

Supplementary Table 2 (ST2): Observed experimental phenomena used to calibrate the model and model performance for each phenomenon.

	Phenomenon	Model
1	$PI(4,5)P_2$ should drop (proportionally) as a consequence of depletion of PI. [5]	A 50% decrease in $_{\rightarrow 0}$ will decrease PI to 50%. This will only cause $PI(4,5)P_2$ to decrease to 88% of steady state. (Fig. 3a)
2	$PI(4,5)P_2$ is independent of PI(4)P. [11] [30]	Even with PI(4)P at 7% of its original steady state levels, $PI(4,5)P_2$ only dropped 20%. (Fig. 3b)
3	$PI(4,5)P_2$ is dependent on PI4K [30]. PI4K knock-down decreases PI(4)P and PI(4,5)P2 by 50%. [11]	A decrease of 95% in PI4K will decrease $PI(4,5)P_2$ to 45% and PI(4)P to 24% of original steady state values. (Fig. 3a)
4	PI(5)P is of similar or higher abundance as compared to PI(3)P and ~20-100-fold below the levels of PI(4)P and $PI(4,5)P_2$. Steady-state PI(5)P levels are more than 5-fold higher than those of $PI(3,5)P_2$. [11,13,15,26,27,29]	PI(5)P = 100.49 PI(3)P = 100.00 PI(4)P = 10040.13 (10040.13 / 100.49 = 99.91173) $PI(4,5)P_2$ = 10000.97 (10000.97 / 100.49 = 99.52204) $PI(3,5)P_2$ = 20.21 (100.49 / 20.21 = 4.972) (Table ST1)
5	Golgi PI(4)P makes a sizeable contribution to the plasma membrane supply of PI(4)P but it is dispensable in the maintenance of $PI(4,5)P_2$. [31]	Closing $\gamma_{\rightarrow 4}$ will decrease PI(4)P to 61% of the steady-state values but only decrease $PI(4,5)P_2$ by 1% (Fig. 3d).
6	$PI(3,4,5)P_3$ depend on PTEN concentration. [13,32]	PI(3,4,5)P3 increases 16 fold when PTEN is knocked down (Fig. 5)
7	PTEN knockdown does not decrease the levels of $PI(4,5)P_2$ significantly. [33]	PI(4,5)P2 decreases 1% when PTEN is knocked down (Fig. 5)
8	$PI(4,5)P_2$ levels drop around 50% if PIP5KI is knocked down 50%. [34]	A decrease in 50% on PIP5KI will decrease $PI(4,5)P_2$ to 55% of the steady-state (Fig. 3a)
9	When PIKfyve is reduced to 10%, PI(5)P and $PI(3,5)P_2$ decrease 50%. [15]	When PIKfyve is reduced to 10%, PI(5)P and $PI(3,5)P_2$ decrease to 50.05% and 49.70% respectively (Fig. 3c).
10	When PIKfyve is reduced to	When PIKfyve is reduced to 0.1%, PI(3)P will

	undetectable levels PI(3)P rises 5 fold, PI(5)P decreases to 15%, PI(3,5)P_2 is undetectable as well and PI(4,5)P_2 decreases to 67-80%. [15]	increase 8.85 fold, PI(5)P decreases to 10.08%, PI(3,5)P_2 to 0.88% and PI(4,5)P_2 to 85.78% of original steady state levels (Fig. 3c).
11	When MTMR2 is knocked out, PI(5)P is reduced to 20% and PI(3,5)P_2 increases to 150%. [15]	When MTMR is reduced to 65% (simulating the knockout of MTMR2), PI(5)P is only reduced to 98.97% but PI(3,5)P_2 is increased to 152.17% (Fig. 3c).
12	Majority of the PI(5)P pool is created from PI(3,5)P_2 via myotubularins. [15]	Three fluxes supply material to the PI(5)P pool: $v_{0\rightarrow5}$ = 5.49 molecules/s, $v_{45\rightarrow5}$ = 17.044 molecules/s and $v_{35\rightarrow5}$ = 102.003 molecules/s. $v_{35\rightarrow5}$, which is catalysed by myotubularins, is responsible for 81.91% of PI(5)P production (Fig. 3c).
13	PI(5)P is important in the synthesis of PI(4,5)P_2. [15]	The flux $v_{5\rightarrow45}$ alone is capable of maintaining PI(4,5)P_2 with 34% of its original steady state levels (Fig. 3b).

Supplementary Table 3 (ST3): Values of model parameters.

Fluxes $v_{i \to j}$	$\gamma_{i \to j}$ (molecules^{1-g} µm^{2g}/min or µm^2/min/mg) where ^{g} is the kinetic order of the corresponding variable	$f_{i \to j}$ (dimensionless)	$E_{i \to j}$ (mg)
$v_{0 \to 3}$	1.09e+15	0.11	2.23E-14
$v_{3 \to 0}$	3.07e+12	1.00	2.56E-14
$v_{0 \to 4}$	5.10e+14	0.29	2.78E-14
$v_{4 \to 0}$	6.65e+12	0.91	8.93E-15
$v_{0 \to 5}$	2.87e+14	0.06	8.54E-15
$v_{5 \to 0}$	3.07e+12	1.00	8.93E-15
$v_{3 \to 35}$	1.34e+14	1.00	8.54E-15
$v_{35 \to 3}$	3.06e+12	1.00	1.71E-13
$v_{4 \to 45}$	8.49e+15	0.05	1.74E-14
$v_{45 \to 4}$	6.65e+12	0.91	6.35E-15
$v_{4 \to 34}$	1.04e+13	0.50	1.23E-15
$V_{34 \to 4}$	7.60e+12	1.00	5.30E-17
$v_{5 \to 45}$	2.95e+13	0.88	6.89E-14
$v_{45 \to 5}$	4.13e+12	0.65	1.05E-14
$v_{35 \to 5}$	4.16e+14	1.00	1.21E-14
$v_{34 \to 3}$	1.33e+12	1.00	3.81E-14
$v_{0 \to 45}$	2.67e+14	0.29	4.15E-14
$V_{45 \to 0}$	6.65e+12	0.91	2.79E-18
$v_{45 \to 345}$	1.89e+14	0.31	1.53E-14
$v_{345 \to 45}$	4.81e+14	0.98	5.30E-17
$v_{345 \to 34}$	1.68e+12	1.00	7.76E-15
$v_{\to 0}$	16588.74	-	-
$v_{\to 3}$	12.37	-	-
$v_{\to 4}$	297.26	-	-
$v_{0 \to}$, $v_{3 \to}$, $v_{4 \to}$, $v_{5 \to}$, $v_{34 \to}$, $v_{35 \to}$, $v_{45 \to}$, $v_{345 \to}$ ($v_{i \to}$)	0.0519	-	-

Supplementary Table 4 (ST4): Kinetic parameters associated with enzymes in the model. The first column contains the enzyme name and the second the corresponding gene name. The third and fourth columns exhibit K_m and specific activity values, along with literature references. The last columns exhibits the localization of the enzyme. Abbreviations: PLIP: PTEN-like lipid phosphatase; TPIP: PTEN-Like Inositol Lipid Phosphatase; N: Nucleus, C: Cytosol; M: Mitochondria; G: Golgi; E: Endosome; ER: Endoplasmic Reticulum; PM: Plasma Membrane.

Protein (EC number)	Human gene name	K_m (mM)	Specific activity (µmol/min/mg)	Localization
PI3K IA α (2.7.1.137)	PIK3CA	0.011 [35]	0.0086 [36]	C, PM
PI3K IA β (2.7.1.137)	PIK3CB		0.0017 [36]	N, C, PM
PI3K IA δ (2.7.1.137)	PIK3CD		0.0026 [36]	C, PM
PI3K IB γ (2.7.1.137)	PIK3CG		0.0016 [36]	C, PM
PI3KII α (2.7.1.154)	PIK3C2A	0.25 [37]	-	N, C, G, PM
PI3KII β (2.7.1.154)	PIK3C2B		-	N, C, ER, E, PM
PI3KII γ (2.7.1.154)	PIK3C2G		-	C, G, PM
PI3KIII (2.7.1.137)	PIK3C3	0.064 [37]	-	C, E
PI4KII α (2.7.1.67)	PI4K2A	0.2 [38]	0.044 [39]	M, E, G, PM
PI4KII β (2.7.1.67)	PI4K2B			N, C, G, E, PM
PI4KIII α (2.7.1.67)	PIK4CA	-	-	C, G, PM
PI4KIII β (2.7.1.67)	PIK4CB	-	-	C, M, ER, G
PIPK I α (2.7.1.68)	PIP5K1A	0.0012 [14]	0.023 [14]	N, C, PM
PIPK I β (2.7.1.68)	PIP5K1B	0.262 [40]		C
PIPK I γ (2.7.1.68)	PIP5K1C	-		N, C, PM
PIPK II α (2.7.1.68)	PIP4K2A	0.05 [40]	0.015 [14]	N, C, PM
PIPK II β (2.7.1.68)	PIP4K2B	-		N, C, ER, PM
PIPK II γ (2.7.1.68)	PIP4K2C	-		C, PM
PIPK III (2.7.1.68)	PIP5K3	-	-	C, G, E
PTEN (3.1.3.67)	PTEN	0.07 [41]	30.318 [41]	N, M, C, PM
TPIP (3.1.3.67)	TPTE2	-	-	C, G, ER
MTM1 (3.1.3.64)	MTM1	0.039 or 0.017 [42]	-	C, E, PM
MTMR1 (3.1.3.64)	MTMR1	0.0008 or 0.0037 [43]	-	C, PM
MTMR2 (3.1.3.64)	MTMR2		-	C, E, PM
MTMR3 (3.1.3.64)	MTMR3		-	C
MTMR4 (3.1.3.64)	MTMR4		-	C, E
MTMR6 (3.1.3.64)	MTMR6		-	N, C
MTMR7 (3.1.3.64)	MTMR7		-	C
MTMR8 (3.1.3.64)	MTMR8		-	N, C
MTMR14 (3.1.3.64)	MTMR14		-	C
INPP4A (3.1.3.66)	INPP4A	0.049 [44]	-	C, E, PM
INPP4B (3.1.3.66)	INPP4B		-	C
TMEM55A (3.1.3.78)	TMEM55A	0.046 [44]	0.01 [45]	C, E
TMEM55B (3.1.3.78)	TMEM55B			N, C, E
SYNJ1 (3.1.3.36)	SYNJ1	0.25 [46 47]	0.765 [47]	C
SYNJ2 (3.1.3.36)	SYNJ2			C, PM
OCRL1 (3.1.3.36)	OCRL			N, C, G, E, PM
INPP5B (3.1.3.36)	INPP5B			C, G, ER, E, PM
INPP5J (3.1.3.36)	INPP5J			C, PM
SKIP (3.1.3.36)	SKIP			N, C, G, ER, PM
SHIP1 (3.1.3.36)	INPP5D			C, PM
SHIP2 (3.1.3.36)	INPPL1			C, G, PM
INPP5E (3.1.3.36)	INPP5E			C, G, PM
SAC1 (3.1.3.36)	SACM1L			G, ER
SAC2 (3.1.3.36)	INPP5F			E
SAC3 (3.1.3.36)	FIG4			G, ER, E

Supplementary Table 5 (ST5): Groups of kinases associated with each flux in the model.

Group	Enzymes	Flux	Values	Reference
pi_3KII_III	PI3KII α / β / γ,<br>PI3K III (Vps34), (primary)<br>PI3K IA α / β / δ,<br>PI3K IB γ (secondary)	$v_{0\rightarrow 3}$	K_m = 0.064<br>SA = 0.0086	[37]<br>[36]
pi_4K	PI4KII α / β,<br>PI4KIII α / β	$v_{0\rightarrow 4}$	K_m = .2<br>SA = 0.044	[38]<br>[48]
pi_Kfyve	PIPK III (secondary)	$v_{0\rightarrow 5}$	K_m = 0.0034<br>No from humans. Bus Taurus.<br>SA = 0.0028<br>(both from PI3K)	[49]<br><br>[36]
pi_Kfyve	PIPK III (primary)	$v_{3\rightarrow 35}$	K_m = 0.12<br>SA estimated	[40]<br>[50]
pi_3KII	PI3K IA α / β / δ,<br>PI3K IB γ,<br>PI3KII α / β / γ<br>(all secondary)	$v_{4\rightarrow 34}$	K_m = 0.25<br>SA = 0.0043 (estimated)	[37]<br>[51]
pip_5KI	PIPK I α / β / γ	$v_{4\rightarrow 45}$	K_m = 0.0012<br>SA = 0.023	[14]<br>[14]
pip_5KII	PIPK II α / β / γ	$v_{5\rightarrow 45}$	K_m = 0.012<br>(No PI(5)P K_m . Using PIP_5KI K_m and SA.)<br>SA = 0.023	[14]<br><br>[14]
pi_3KI	PI3K IA α / β / δ,<br>PI3K IB γ	$v_{45\rightarrow 345}$	K_m = 0.011<br>SA = 0.0086	[35]<br>[36]
pi_4K_pip_5KI	PI4K, PIP5KI and Dvl	$v_{0\rightarrow 45}$	K_m = .2 (K_m of the first enzyme to contact substrate)<br>SA = 0.023 (SA of the slowest)	[38]<br>[14]

Supplementary Table 6 (ST6): Groups of phosphatases and associated fluxes.

Group	Enzymes	Flux	Values (problems)	Reference
SYNJ_SAC1_MTMR	SYNJ 1/2, SAC1, MTMR (9 forms)	$v_{3\rightarrow 0}$		
SYNJ_SAC1	SYNJ 1/2, SAC1	$v_{4\rightarrow 0}$	K_m = 0.25<br>(Using PI(4,5)P2 K_m of 5 phosphatase)<br>SA = 0.765	46<br>47
SYNJ_SAC1	SYNJ 1/2, SAC1	$v_{5\rightarrow 0}$		
SYNJ_SAC1_SAC3	SYNJ 1/2, SAC1, SAC3	$v_{35\rightarrow 3}$		
SYNJ	Not yet documented. Possibly synaptojanins.	$v_{45\rightarrow 0}$		
S_I_O_S_S	SYNJ 1/2, INPP5 B/J/E, OCRL1, SAC2, SKIP	$v_{45\rightarrow 4}$	K_m = 0.25<br>(Using PI(4,5)P2 K_m of 5 phosphatase)<br>SA = 0.765	46<br>47
S_I_O_S_S_SHIP2	SYNJ 1/2, INPP5 B/J/E, OCRL1, SAC2, SKIP, SHIP2	$v_{345\rightarrow 34}$	K_m = 0.25<br>(Using PI(4,5)P2 K_m of 5 phosphatase)<br>SA = 0.0765<br>(SA estimated)	46
SYNJ_TMEM55	SYNJ 1/2, TMEM55	$v_{45\rightarrow 5}$	K_m = 0.046<br>(Using Inositol134P3 Km)<br>SA = 0.01	44<br>45
PTEN	PTEN, TPTE	$v_{345\rightarrow 45}$	K_m = 0.07<br>SA = 30.3	41<br>41
MTMR	MTMR 7 forms, TPIP, INPP5E	$v_{35\rightarrow 5}$	K_m = 0.0037<br>(Rattus Inositol13P2 K_m.<br>No SA<br>(SA estimated)	43<br>47
INPP4	INPP4 A/B	$v_{34\rightarrow 3}$	K_m = 0.046<br>(Using Inositol134P3 K_m.<br>No SA. Using PI(4,5)P2_4P SA)<br>SA = 0.061	44<br>45
PTEN	not yet documented	$v_{34\rightarrow 4}$	K_m = 0.72<br>SA = 5.47	52<br>52

Supplementary Table 7 (ST7): Performance and Score Criteria for the Genetic Algorithm.

Performance criteria:

- The solution must exist; it must not be composed of NA or NaN values
- The system must reach a stable steady state
- 200000 < PI < 400000
- 0.1< PI(3)P < 6000
- 5000 < PI(4)P < 20000
- 0.1< PI(5)P < 200
- 0.1< PI(3,5)P_2 < 200
- 5000 < PI(4,5)P_2 < 20000
- 0.1< PI(3,4)P_2 < 200
- 0.1< PI(3,4,5)P_3 < 5000
- The sum of all phosphoinositides must be between 220000 and 450000

Score criteria:

- Similar levels of PI(4)P and PI(4,5)P_2
- Similar levels of PI(3)P and PI(5)P
- PI(5)P levels are 5 fold of PI(3,5)P2 levels
- PI(4)P and PI(4,5)P_2 levels are 100 times higher than PI(5)P and PI(3)P
- Steady-state value of PI close to 300000
- Steady-state value of PI(4,5)P_2 close to 10000
- Steady-state value of PI(3)P close to 100
- Similar levels of PI35P_2 and PI34P_2
- PI(4,5)P_2 will decrease by the same percentage as PI
- PI(4)P drops to 50% after PI4K knockout
- PI(4,5)P_2 drops to 50% after PI4K knockout
- PI(4,5)P2 drops to 50% after PIP_5KI knockdown
- PI(5)P drops to 20% after MTMR2 knockdown (MTMR estimated to be reduced to 65%)
- PI(3,5)P_2 raises to 150% after MTMR2 knockdown (MTMR estimated to be reduced to 65%)
- PI(5)P should drop to 50% if pi_Kfive is reduced to 10%
- PI35P_2 should drop to 50% if pi_Kfive is reduced to 10%
- PI(5)P should drop to 15% if pi_Kfyve is knockout
- PI(3,5)P_2 should drop to 0.1% if pi_Kfyve is knockout
- PI(4,5)P_2 should drop to 80% if pi_Kfyve is knockout
- PI(3)P should increase 5-fold if pi_Kfyve is knockout

Supplementary Table 8 (ST8): Local sensitivity analysis. Each parameter was altered by 1% and the consequent alteration in each pool was measured. Induced changes greater than 1% are highlighted.

	PI	PI(3)P	PI(4)P	PI(5)P	PI(3,5)P2	PI(4,5)P2	PI(3,4)P2	PI(3,4,5)P3
$\gamma_{\rightarrow 0}$	**1.03456**	0.10471	0.24441	0.11920	0.10457	0.19251	0.09790	0.05926
$\gamma_{\rightarrow 4}$	0.00570	0.00165	0.38978	0.00224	0.00165	0.00698	0.12026	0.00215
$\gamma_{\rightarrow 3}$	0.00012	0.10674	0.00318	0.09998	0.10661	0.01453	0.00271	0.00448
$\gamma_{0\rightarrow 3}$	-0.00544	0.88524	0.02484	0.82935	0.88412	0.11939	0.02188	0.03676
$f_{0\rightarrow 3}$	-0.00788	**1.28161**	0.03596	**1.20096**	**1.27999**	0.17285	0.03167	0.05321
$\gamma_{3\rightarrow 0}$	0.00042	-0.06751	-0.00189	-0.06322	-0.06743	-0.00911	-0.00167	-0.00281
$f_{3\rightarrow 0}$	0.00195	-0.31694	-0.00889	-0.29675	-0.31655	-0.04275	-0.00784	-0.01317
$\gamma_{0\rightarrow 4}$	-0.02276	-0.00041	0.68316	0.00017	-0.00041	0.00622	0.20991	0.00192
$f_{0\rightarrow 4}$	-0.08381	-0.00155	**2.51637**	0.00057	-0.00155	0.02259	0.76967	0.00696
$\gamma_{4\rightarrow 0}$	0.01114	0.00020	-0.33438	-0.00009	0.00020	-0.00307	-0.10300	-0.00095
$f_{4\rightarrow 0}$	0.09480	0.00164	**-2.84494**	-0.00085	0.00164	-0.02664	-0.88199	-0.00821
$\gamma_{0\rightarrow 5}$	-0.00031	-0.00002	0.00152	0.05046	-0.00002	0.00727	0.00133	0.00224
$f_{0\rightarrow 5}$	-0.00025	-0.00002	0.00123	0.04087	-0.00002	0.00589	0.00108	0.00181
$\gamma_{5\rightarrow 0}$	0.00016	0.00001	-0.00076	-0.02521	0.00001	-0.00363	-0.00067	-0.00112
$f_{5\rightarrow 0}$	0.00073	0.00005	-0.00357	-0.11872	0.00005	-0.01711	-0.00314	-0.00527
$\gamma_{3\rightarrow 35}$	-0.00036	-0.88106	0.00320	0.10443	0.11136	0.01509	0.00279	0.00465
$f_{3\rightarrow 35}$	-0.00164	**-3.98069**	0.01448	0.47192	0.50315	0.06820	0.01259	0.02100
$\gamma_{35\rightarrow 3}$	0.00003	0.08270	-0.00030	-0.00980	-0.01045	-0.00142	-0.00026	-0.00044
$f_{35\rightarrow 3}$	0.00010	0.25230	-0.00092	-0.02990	-0.03189	-0.00432	-0.00080	-0.00133
$\gamma_{4\rightarrow 45}$	-0.00329	-0.00061	-0.22535	0.03145	-0.00061	0.31978	-0.03098	0.09840
$f_{4\rightarrow 45}$	-0.00140	-0.00026	-0.09563	0.01335	-0.00026	0.13571	-0.01313	0.04179
$\gamma_{45\rightarrow 4}$	0.00266	0.00049	0.18242	-0.02548	0.00049	-0.25884	0.02496	-0.07981
$f_{45\rightarrow 4}$	0.02277	0.00418	**1.56142**	-0.21880	0.00418	**-2.21482**	0.21030	-0.68757
$\gamma_{5\rightarrow 45}$	-0.00014	0.00001	0.00257	**-1.04473**	0.00001	0.01195	0.00222	0.00368
$f_{5\rightarrow 45}$	-0.00056	0.00002	0.01020	**-4.15151**	0.00002	0.04751	0.00880	0.01463
$\gamma_{45\rightarrow 5}$	0.00002	0.00000	-0.00038	0.15426	0.00000	-0.00177	-0.00033	-0.00054
$f_{45\rightarrow 5}$	0.00013	-0.00001	-0.00233	0.95018	-0.00001	-0.01087	-0.00202	-0.00335
$\gamma_{45\rightarrow 345}$	-0.00015	0.00340	-0.01062	-0.00175	0.00340	-0.04880	0.38096	0.99045
$f_{45\rightarrow 345}$	-0.00044	0.00973	-0.03037	-0.00501	0.00972	-0.13959	**1.08973**	**2.83323**
$\gamma_{345\rightarrow 45}$	0.00005	-0.00101	0.00316	0.00052	-0.00101	0.01451	-0.11324	-0.29441
$f_{345\rightarrow 45}$	0.00029	-0.00650	0.02027	0.00334	-0.00649	0.09317	-0.72733	**-1.89095**
$\gamma_{35\rightarrow 5}$	-0.00003	-0.08108	0.00059	0.01908	-0.97009	0.00276	0.00051	0.00085
$f_{35\rightarrow 5}$	-0.00008	-0.24026	0.00176	0.05653	**-2.87469**	0.00819	0.00152	0.00252
$\gamma_{34\rightarrow 3}$	0.00000	0.00456	0.00013	0.00427	0.00455	0.00062	-0.48909	0.00019
$f_{34\rightarrow 3}$	0.00001	0.01376	0.00039	0.01289	0.01375	0.00187	**-1.47765**	0.00058
$\gamma_{345\rightarrow 34}$	0.00000	0.00343	0.00003	0.00317	0.00342	0.00012	0.38195	-0.01704
$f_{345\rightarrow 34}$	0.00002	0.02280	0.00020	0.02112	0.02277	0.00078	**2.54154**	-0.11336
$\gamma_{i\rightarrow}$ (i=0,3,4,5,35,34,45,345)	-0.98865	-0.15655	**-1.07621**	-0.30012	-0.16558	-0.96498	**-1.21047**	-0.98028
$\gamma_{0\rightarrow 45}$	-0.02376	-0.00143	0.12280	0.05741	-0.00143	0.58758	0.10772	0.18064
$f_{0\rightarrow 45}$	-0.08750	-0.00528	0.45199	0.21086	-0.00527	**2.16408**	0.39516	0.66172
$\gamma_{4\rightarrow 34}$	-0.00002	0.00549	-0.00153	0.00514	0.00548	0.00072	0.61188	0.00022
$f_{4\rightarrow 34}$	-0.00009	0.02592	-0.00723	0.02426	0.02589	0.00339	**2.89031**	0.00104
$\gamma_{34\rightarrow 4}$	0.00000	-0.00004	0.00001	-0.00003	-0.00004	0.00000	-0.00391	0.00000
$f_{34\rightarrow 4}$	0.00000	-0.00011	0.00003	-0.00010	-0.00011	-0.00001	-0.01202	0.00000
$\gamma_{45\rightarrow 0}$	0.00000	0.00000	-0.00002	-0.00001	0.00000	-0.00011	-0.00002	-0.00004
$f_{45\rightarrow 0}$	0.00004	0.00000	-0.00021	-0.00010	0.00000	-0.00100	-0.00018	-0.00031

Supplementary Table 9 (ST9): Phenomena used to train the model, formulae used to evaluate the model for each phenomenon, and respective non-normalized scores.

Data	Formula to calculate non-normalized score	non-normalized score
Similar levels of PI4P and PI45P2	abs([PI(4)P] - [PI(4,5)P2])	617.815
Similar levels of PI3P and PI5P	abs([PI(3)P] - [PI(5)P])	17.86241
PI5P levels are 5 fold of PI35P2 levels	abs([PI(5)P] - 5 * [PI(3,5)P2])	7.64199
PI4P or PI45P2 is 100 times more than PI5P or PI3P	abs([PI(4)P] - 100 * [PI(5)P])	930.429
Stst value of PI close to 300000	abs([PI] - 300000)	19618.6
Stst value of PI45P2 close to 10000	abs([PI(4,5)P2] - 10000)	519.085
Stst value of PI3P close to 100	abs([PI(3)P] - 100)	7.57082
similar levels of PI35P2 and PI34P2	abs([PI(3,5)P2] - [PI(3,4)P2])	17.05115
PI45P2 will decrease in the same percentage as PI	abs([PI with PI input KD] / [Steady-state PI] - [PI(4,5)P2 with PI input KD] / [Steady-state PI(4,5)P2])	0.4633076
PI4P drops to .5 after PI4K knockout	abs(0.5 - [PI(4)P with PI4K KO] / [Steady-state PI(4)P])	0.4111381
PI45P2 drops to .5 after PI4K knockout	abs(0.5 - [PI(4,5)P2 with PI4K KO] / [Steady-state PI(4,5)P2])	0.3112194
PI45P2 drops to .5 after PIP_5KI knockdown	abs(0.5 - [PI(4,5)P2 with PIP5KI KD] / [Steady-state PI(4,5)P2])	0.03934597
PI5P drops to .2 after MTMR2 knockdown (MTMR estimated to be reduced to 65%)	abs(0.2 - [PI(5)P with MTMR 35% KD] / [Steady-state PI(5)P])	0.7106936
PI35P2 raises to 1.5 after MTMR2 knockdown (MTMR estimated to be reduced to 65%)	abs(1.5 - [PI(3,5)P2 with MTMR 35% KD] / [Steady-state PI(3,5)P2])	0.02895678
PI5P should drop to 50% if pi_Kfive is reduced to 10%	abs(0.5 - [PI(5)P with PIKfyve 90% KD] / [Steady-state PI(5)P])	0.06829588
PI35P2 should drop to 50% if pi_Kfive is reduced to 10%	abs(0.5 - [PI(3,5)P2 with PIKfyve 90% KD] / [Steady-state PI(3,5)P2])	0.02601925
PI5P should drop to .15 if pi_Kfyve is knockout	abs(0.15 - [PI(5)P with PIKfyve KO] / [Steady-state PI(5)P])	0.07319392
PI35P2 should drop to undetectable levels if pi_Kfyve is knockout	abs(0.001 - [PI(3,5)P2 with PIKfyve KO] / [Steady-state PI(3,5)P2])	0.007045735
PI45P2 should drop to .8 if pi_Kfyve is knockout	abs(0.8 - [PI(4,5)P2 with PIKfyve KO] / [Steady-state PI(4,5)P2])	0.04462109
PI3P should increase 5-fold if pi_Kfyve is knockout	abs(5 - [PI(3)P with PIKfyve KO] / [Steady-state PI(3)P])	3.074068

Supplementary Table 10 (ST10): siRNAs screen used to identify candidate CF drug targets. Effect of siRNA is measured by ENaC activity. Scores above 1 classify a gene as ENaC inhibiting and below -1 as ENaC-activating. The screen is described in detail in theses supplementary methods and in Almaça *et al.* [23].

First round of siRNA screens (*Almaça et al.* [23])		Second round of siRNA screens (siRNA KD validation tests)				
Gene name	Results	Gene name	First siRNA test	Second siRNA test	Third siRNA test	Results
INPP5A	ENaC-activating gene	INPP5A	4.628	-2.796	x	both effects
INPPL1	ENaC-activating gene	INPPL1	3.400	-1.025	x	both effects
OCRL	both effects	OCRL	1.947	0.465	-4.844	both effects
PI4KA	ENaC-activating gene	PI4KA	-0.861	-5.739	4.752	both effects
PIK3C2B	ENaC-inhibiting gene	PIK3C2B	5.777	-0.019	x	1 of 2 ENaC-inhibiting
PIK3CA	ENaC-activating gene	PIK3CA	-1.761	1.109	-1.385	both effects
PIK3CB	ENaC-activating gene	PIK3CB	-0.509	-4.154	x	1 of 2 ENaC-activating
PIK3CD	ENaC-activating gene	PIK3CD	-2.944	-1.903	0.952	2 of 3 ENaC-activating
PIK3R3	ENaC-inhibiting gene	PIK3R3	5.877	-11.030	2.551	both effects
PIP4K2A	ENaC-activating gene	PIP4K2A	-2.992	-4.393	-3.249	3 of 3 ENaC-activating
PIP5K1B	both effects	PIP5K1B	-1.986	-3.728	x	2 of 2 ENaC-activating

Supplementary Table 11 (ST11). System equations.

Fluxes

$V_{\rightarrow 0} = \gamma_{\rightarrow 0}$

$V_{\rightarrow 4} = \gamma_{\rightarrow 4}$

$V_{\rightarrow 3} = \gamma_{\rightarrow 3}$

$V_{0\rightarrow 3} = \gamma_{0\rightarrow 3} * \text{PI}^\wedge f_{0\rightarrow 3} * (\text{PI3KII+PI3III})$

$V_{3\rightarrow 0} = \gamma_{3\rightarrow 0} * \text{PI(3)P}^\wedge f_{3\rightarrow 0} * (\text{SYNJ+SAC1+MTMR})$

$V_{0\rightarrow 4} = \gamma_{0\rightarrow 4} * \text{PI}^\wedge f_{0\rightarrow 4} * \text{PI4K}$

$V_{4\rightarrow 0} = \gamma_{4\rightarrow 0} * \text{PI(4)P}^\wedge f_{4\rightarrow 0} * (\text{SYNJ+SAC1})$

$V_{0\rightarrow 5} = \gamma_{0\rightarrow 5} * \text{PI}^\wedge f_{0\rightarrow 5} * \text{PIKfyve}$

$V_{5\rightarrow 0} = \gamma_{5\rightarrow 0} * \text{PI(5)P}^\wedge f_{5\rightarrow 0} * (\text{SYNJ+SAC1})$

$V_{3\rightarrow 35} = \gamma_{3\rightarrow 35} * \text{PI(3)P}^\wedge f_{3\rightarrow 35} * \text{PIKfyve}$

$V_{35\rightarrow 3} = \gamma_{35\rightarrow 3} * \text{PI(3,5)P}_2{}^\wedge f_{35\rightarrow 3} * (\text{SYNJ+SAC1+SAC3})$

$V_{4\rightarrow 45} = \gamma_{4\rightarrow 45} * \text{PI(4)P}^\wedge f_{4\rightarrow 45} * \text{PIP5KI}$

$V_{45\rightarrow 4} = \gamma_{45\rightarrow 4} * \text{PI(4,5)P2}^\wedge f_{45\rightarrow 4} * (\text{SIOSS})$

$V_{5\rightarrow 45} = \gamma_{5\rightarrow 45} * \text{PI(5)P}^\wedge f_{5\rightarrow 45} * \text{PIP5KII}$

$V_{45\rightarrow 5} = \gamma_{45\rightarrow 5} * \text{PI(4,5)P}_2{}^\wedge f_{45\rightarrow 5} * (\text{SYNJ+TMEM55})$

$V_{45\rightarrow 345} = \gamma_{45\rightarrow 345} * \text{PI(4,5)P}_2{}^\wedge f_{45\rightarrow 345} * \text{PI3KI}$

$V_{345\rightarrow 45} = \gamma_{345\rightarrow 45} * \text{PI(3,4,5)P}_3{}^\wedge f_{345\rightarrow 45} * \text{PTEN}$

$V_{35\rightarrow 5} = \gamma_{35\rightarrow 5} * \text{PI(3,5)P}_2{}^\wedge f_{35\rightarrow 5} * \text{MTMR}$

$V_{34\rightarrow 3} = \gamma_{34\rightarrow 3} * \text{PI(3,4)P}_2{}^\wedge f_{34\rightarrow 3} * \text{INPP4}$

$V_{345\rightarrow 34} = \gamma_{345\rightarrow 34} * \text{PI(3,4,5)P}_3{}^\wedge f_{345\rightarrow 34} * (\text{SIOSS+SHIP2})$

$V_{45\rightarrow} = \gamma_{45\rightarrow} * \text{PI(4,5)P}_2$

$V_{0\rightarrow} = \gamma_{0\rightarrow} * \text{PI}$

$V_{4\rightarrow} = \gamma_{4\rightarrow} * \text{PI(4)P}$

$V_{345\rightarrow} = \gamma_{345\rightarrow} * \text{PI(3,4,5)P}_3$

$V_{3\rightarrow} = \gamma_{3\rightarrow} * \text{PI(3)P}$

$V_{35\rightarrow} = \gamma_{35\rightarrow} * \text{PI(3,5)P}_2$

$V_{5\rightarrow} = \gamma_{5\rightarrow} * \text{PI(5)P}$

$V_{34\rightarrow} = \gamma_{34\rightarrow} * \text{PI(3,4)P}_2$

$V_{0\rightarrow 45} = \gamma_{0\rightarrow 45} * \text{PI}^\wedge f_{0\rightarrow 45} * (\text{PI4K+PIP5KI})$

$V_{4\rightarrow 34} = \gamma_{4\rightarrow 34} * \text{PI(4)P}^\wedge f_{4\rightarrow 34} * \text{PI3KII}$

$V_{34\rightarrow 4} = \gamma_{34\rightarrow 4} * \text{PI(3,4)P}_2{}^\wedge f_{34\rightarrow 4} * \text{PTEN}$

$V_{45\rightarrow 0} = \gamma_{45\rightarrow 0} * \text{PI(4,5)P}_2{}^\wedge f_{45\rightarrow 0} * \text{SYNJ}$

Differential equations

$dPI = V_{\rightarrow 0} + V_{3\rightarrow 0} + V_{4\rightarrow 0} + V_{5\rightarrow 0} + V_{45\rightarrow 0} - V_{0\rightarrow 3} - V_{0\rightarrow 4} - V_{0\rightarrow 5} - V_{0\rightarrow 45} - V_{0\rightarrow}$

$dPI3P = V_{\rightarrow 3} + V_{0\rightarrow 3} + V_{35\rightarrow 3} + V_{34\rightarrow 3} - V_{3\rightarrow 0} - V_{3\rightarrow 35} - V_{3\rightarrow}$

$dPI4P = V_{\rightarrow 4} + V_{0\rightarrow 4} + V_{45\rightarrow 4} + V32 - V_{4\rightarrow 0} - V_{4\rightarrow 45} - V24 - V_{4\rightarrow}$

$dPI5P = V_{0\rightarrow 5} + V_{35\rightarrow 5} + V_{45\rightarrow 5} - V_{5\rightarrow 0} - V_{5\rightarrow 45} - V_{5\rightarrow}$

$dPI35P2 = V_{3\rightarrow 35} - V_{35\rightarrow 5} - V_{35\rightarrow 3} - V_{35\rightarrow}$

$dPI45P2 = V_{4\rightarrow 45} + V_{5\rightarrow 45} + V_{345\rightarrow 45} + V_{0\rightarrow 45} - V_{45\rightarrow 4} - V_{45\rightarrow 5} - V_{45\rightarrow 345} - V_{45\rightarrow 0} - V_{45\rightarrow}$

$dPI34P2 = V_{345\rightarrow 34} + V_{4\rightarrow 34} - V_{34\rightarrow 4} - V_{34\rightarrow 3} - V_{34\rightarrow}$

$dPI345P3 = V_{45\rightarrow 345} - V_{345\rightarrow 45} - V_{345\rightarrow 34} - V_{345\rightarrow}$

4. REFERENCES

1. van Meer, G., Voelker, D. R. & Feigenson, G. W. Membrane lipids: where they are and how they behave. *Nat. Rev. Mol. Cell Biol.* **9,** 112–124 (2008).

2. Fadeel, B. & Xue, D. Membrane : Roles in Health and Disease. *Crit Rev Biochem Mol Biol* **44,** 264–277 (2009).

3. Phillips, M. J. & Voeltz, G. K. Structure and function of ER-membrane contact sites with other organelles. *Mol. Cell Biol.* **17,** 69–82 (2016).

4. Lev, S. Non-vesicular lipid transport by lipid-transfer proteins and beyond. *Nat. Rev. Mol. Cell Biol.* (2010). doi:10.1038/nrm2971

5. Kim, Y. J., Guzman-Hernandez, M. L. & Balla, T. A highly dynamic ER-derived phosphatidylinositol-synthesizing organelle supplies phosphoinositides to cellular membranes. *Dev. Cell* **21,** 813–824 (2011).

6. Johnson, A. *et al.* Molecular Biology of the Cell. (4th ed.). *Garland Science* Visualized in 2016. Available from: https://www.nc (2002).

7. Schomburg, I. *et al.* BRENDA: integrated reactions, kinetic data, enzyme function data, improved disease classification. Retrieved 2015, from http://www.brenda-enzymes.org (2015).

8. Chen, P. W., Fonseca, L. L., Hannun, Y. A. & Voit, E. O. Coordination of Rapid Sphingolipid Responses to Heat Stress in Yeast. *PLoS Comput. Biol.* **9,** (2013).

9. Sasaki, T. *et al.* Mammalian phosphoinositide kinases and phosphatases. *Prog. Lipid Res.* **48,** 307–343 (2009).

10. Stefan, C. J. *et al.* Osh proteins regulate phosphoinositide metabolism at ER-plasma membrane contact sites. *Cell* **144,** 389–401 (2011).

11. Balla, T. Phosphoinositides: tiny lipids with giant impact on cell regulation. *Physiol. Rev.* **93,** 1019–1137 (2013).

12. Delage, E., Puyaubert, J., Zachowski, A. & Ruelland, E. Signal transduction pathways involving phosphatidylinositol 4-phosphate and phosphatidylinositol 4,5-bisphosphate: Convergences and divergences among eukaryotic kingdoms. *Prog. Lipid Res.* **52,** 1–14 (2013).

13. Leslie, N. R., Batty, I. H., Maccario, H., Davidson, L. & Downes, C. P. Understanding PTEN regulation: PIP2, polarity and protein stability. *Oncogene* **27,** 5464–5476 (2008).

14. Bazenet, C. E. & Anderson, R. A. Phosphatidylinositol-4-phosphate 5-kinases from human erythrocytes. *Methods Enzym.* **209,** 189–202 (1992).

15. Bulley, S. J., Clarke, J. H., Droubi, A., Giudici, M.-L. & Irvine, R. F. Exploring phosphatidylinositol 5-phosphate 4-kinase function. *Adv. Biol. Regul.* **57,** 193–202 (2015).

16. Emerling, B. M. *et al.* Depletion of a putatively druggable class of phosphatidylinositol kinases inhibits growth of p53-Null tumors. *Cell* **155,** 844–857 (2013).

17. Schaletzky, J. *et al.* Phosphatidylinositol-5-Phosphate Activation and Conserved Substrate Specificity of the Myotubularin Phosphatidylinositol 3-Phosphatases. *Curr. Biol.* **13,** 504–509 (2003).

18. Divecha, N. Phosphoinositides in the nucleus and myogenic differentiation: how a nuclear turtle with a PHD builds muscle. *Biochem. Soc. Trans.* **44,** 299–306 (2016).

19. Kent, E., Neumann, S., Kummer, U. & Mendes, P. What can we learn from global sensitivity analysis of biochemical systems? *PLoS One* **8,** (2013).

20. Srinath, S. & Gunawan, R. Parameter identifiability of power-law biochemical system models. *J. Biotechnol.* **149,** 132–140 (2010).

21. Yao, K. Z., Shaw, B. M., Kou, B., McAuley, K. B. & Bacon, D. W. Modeling Ethylene/Butene Copolymerization with Multi-site Catalysts: Parameter Estimability and Experimental Design. *Polym. React. Eng.* **11,** 563–588 (2003).

22. R Core Team. *R: A Language and Environment for Statistical Computing*. (2017).

23. Almaça, J. *et al.* High-content siRNA screen reveals global ENaC regulators and potential cystic fibrosis therapy targets. *Cell* **154,** (2013).

24. Xu, C., Watras, J. & Loew, L. M. Kinetic analysis of receptor-activated phosphoinositide turnover. *J. Cell Biol.* **161,** 779–791 (2003).

25. Viaud, J. *et al.* Phosphoinositides: Important lipids in the coordination of cell dynamics. *Biochimie* **125,** 250–258 (2016).

26. Ikonomov, O. C. *et al.* The phosphoinositide kinase PIKfyve is vital in early embryonic development: Preimplantation lethality of PIKfyve-/- embryos but normality of PIKfyve+/- mice. *J. Biol. Chem.* **286,** 13404–13413 (2011).

27. Zhang, Y. *et al.* Loss of Vac14, a regulator of the signaling lipid phosphatidylinositol 3,5-bisphosphate, results in neurodegeneration in mice. *Proc. Natl. Acad. Sci. U. S. A.* **104,** 17518–17523 (2007).

28. Falkenburger, B. H., Jensen, J. B., Dickson, E. J., Suh, B.-C. & Hille, B. Phosphoinositides: lipid regulators of membrane proteins. *J. Physiol.* (2010). doi:10.1113/jphysiol.2010.192153

29. Shisheva, A. PtdIns5P: News and views of its appearance, disappearance and deeds. *Arch. Biochem. Biophys.* **538,** 171–180 (2013).

30. Hammond, G. R. V. *et al.* PI4P and PI(4,5)P2 Are Essential But Independent Lipid Determinants of Membrane Identity. *Science (80-.).* **337,** 727–730 (2012).

31. Szentpetery, Z., Várnai, P. & Balla, T. Acute manipulation of Golgi phosphoinositides to assess their importance in cellular trafficking and signaling. *Proc. Natl. Acad. Sci. U. S. A.* **107,** 8225–30 (2010).

32. Martin-Belmonte, F. *et al.* PTEN-Mediated Apical Segregation of Phosphoinositides

Controls Epithelial Morphogenesis through Cdc42. *Cell* (2007). doi:10.1016/j.cell.2006.11.051

33. Gericke, A., Leslie, N. R., Lösche, M. & Ross, A. H. PtdIns(4,5)P2-mediated cell signaling: Emerging principles and PTEN as a paradigm for regulatory mechanism. *Adv. Exp. Med. Biol.* **991,** 85–104 (2013).

34. Volpicelli-Daley, L. A. *et al.* Phosphatidylinositol-4-phosphate 5-kinases and phosphatidylinositol 4,5-bisphosphate synthesis in the brain. *J. Biol. Chem.* **285,** 28708–28714 (2010).

35. Jones, D. R. *et al.* Phosphorylation of glycosyl-phosphatidylinositol by phosphatidylinositol 3-kinase changes its properties as a substrate for phospholipases. *FEBS Lett., 579(1), 59-65.* **579,** 59–65 (2005).

36. Meier, T. I. *et al.* Cloning, expression, purification, and characterization of the human Class Ia phosphoinositide 3-kinase isoforms. Protein Expr Purif. *Protein Expr Purif.* **35,** 218–224 (2004).

37. Domin, J. *et al.* Cloning of a human phosphoinositide 3-kinase with a C2 domain that displays reduced sensitivity to the inhibitor wortmannin. *Biochem J.* **326,** 139–147. (1997).

38. Wetzker, R. *et al.* Purification and characterization of phosphatidylinositol 4-kinase from human erythrocyte membranes. *Eur J Biochem.* **200,** 179–185 (1991).

39. Jenkins, G. H. & Subrahmanyam, G. Anderson, R. A. Purification and reconstitution of phosphatidylinositol 4-kinase from human erythrocytes. *Biochim Biophys Acta.* **1080,** 11–28 (1991).

40. Zhang, X. *et al.* Phosphatidylinositol-4-phosphate 5-kinase isozymes catalyze the synthesis of 3-phosphate-containing phosphatidylinositol signaling molecules. *J Biol Chem.* **272,** 17756–17761 (1997).

41. Johnston, S. B. & Raines, R. T. Catalysis by the tumor-suppressor enzymes PTEN and PTEN-L. *PLoS One* **10,** 1–13 (2015).

42. Consortium, T. U. Uniprot : a hub for Protein information. *The UniProt Consortium.* Retrieved October 21, 2015, from http://www.unipro (2015).

43. Cladwell, K. K., Lips, D. L., Bansal, V. S. & Majerus, P. W. Isolation and characterization of two 3-phosphatases that hydrolyze both phosphatidylinositol 3-phosphate and inositol 1,3-bisphosphate. *J Biol Chem* **266,** 18378–18386 (1991).

44. Norris, F. A., Atkins, R. C. & Majerus, P. W. The cDNA cloning and characterization of inositol polyphosphate 4-phosphatase type II. Evidence for conserved alternative splicing in the 4-phosphatase family. *J Biol Chem.* **272,** 23859–23864 (1997).

45. Niebuhr, K. *et al.* Conversion of PtdIns(4,5)P(2) into PtdIns(5)P by the S.flexneri effector IpgD reorganizes host cell morphology. *EMBO J.* **21,** 5069–5078 (2002).

46. Matzaris, M., Jackson, S. P., Laxminarayan, K. M., Speed, C. J. & Mitchell, C. A. Identification and characterization of the phosphatidylinositol-(4, 5)-bisphosphate 5-phosphatase in human platelets. *J Biol Chem.* **269,** 3397–3402 (1994).

47. Roach, P. D. & Palmer, F. B. Human erythrocyte cytosol phosphatidyl-inositol-bisphosphate phosphatase.Human erythrocyte cytosol phosphatidyl-inositol-bisphosphate phosphatase. *Biochim Biophys Acta.* 323–333 (1981).

48. Suer, S., Sickmann, A., Meyer, H. E., Herberg, F. W. & Heilmeyer Jr., L. M. Human phosphatidylinositol 4-kinase isoform PI4K92. Expression of the recombinant enzyme and determination of multiple phosphorylation sites. *Eur J Biochem.* **268,** 2099–2106 (2001).

49. Morgan, S. J., Smith, A. D. & Parker, P. J. Purification and characterization of bovine brain type I phosphatidylinositol kinase. *Eur. J. Biochem.* **191,** 761–767 (1990).

50. Ling, L. E., Schultz, J. T. & Cantley, L. C. Characterization and purification of membrane-associated phosphatidylinositol-4-phosphate kinase from human red blood cells. *J. Biol. Chem.* **264,** 5080–5088 (1989).

51. Carpenter, C. L. *et al.* Purification and characterization of phosphoinositide 3-kinase from rat liver. *J Biol Chem.* **265,** 19704–19711 (1990).

52. McConnachie, G., Pass, I. & Walker, S. M. Interfacial kinetic analysis of the tumour suppressor phosphatase, PTEN: evidence for activation by anionic phospholipids. *Biochem. J.* **371,** 947–955 (2003).
